# The genome sequence of the Rose-flounced Tabby, *Endotricha flammealis *(Denis & Schiffermüller, 1775)

**DOI:** 10.12688/wellcomeopenres.19923.1

**Published:** 2023-11-13

**Authors:** Douglas Boyes, Kaouthar Eljounaidi, University of Oxford and Wytham Woods Genome Acquisition Lab, Darwin Tree of Life Barcoding collective, Wellcome Sanger Institute Tree of Life programme, Wellcome Sanger Institute Scientific Operations: DNA Pipelines collective, Tree of Life Core Informatics collective, Darwin Tree of Life Consortium

**Affiliations:** 1 UK Centre for Ecology & Hydrology, Wallingford, England, UK; 2 Centre for Novel Agricultural Products (CNAP), University of York, York, England, UK

**Keywords:** Endotricha flammealis, Rose-flounced Tabby, genome sequence, chromosomal, Lepidoptera

## Abstract

We present a genome assembly from an individual female *Endotricha flammealis* (the Rose-flounced Tabby; Arthropoda; Insecta; Lepidoptera; Pyralidae). The genome sequence is 473.9 megabases in span. Most of the assembly is scaffolded into 32 chromosomal pseudomolecules, including the W and Z sex chromosomes. The mitochondrial genome has also been assembled and is 15.23 kilobases in length. Gene annotation of this assembly on Ensembl identified 17,578 protein coding genes.

## Species taxonomy

Eukaryota; Metazoa; Eumetazoa; Bilateria; Protostomia; Ecdysozoa; Panarthropoda; Arthropoda; Mandibulata; Pancrustacea; Hexapoda; Insecta; Dicondylia; Pterygota; Neoptera; Endopterygota; Amphiesmenoptera; Lepidoptera; Glossata; Neolepidoptera; Heteroneura; Ditrysia; Obtectomera; Pyraloidea; Pyralidae; Pyralinae; *Endotricha*; *Endotricha flammealis* (Denis & Schiffermüller, 1775) (NCBI:txid1101095).

## Background

*Endotricha flammealis,* also known as the Rose-flounced Tabby, is a species of snout moth of the family Pyralidae. This species is found in western, central, and southern Europe, extending to Turkey, Crimea, Cyprus, Iran, Lebanon, Syria, and north Africa’s Maghreb region, which includes Algeria and Tunisia. While it is widespread in southern Britain, it is absent in Ireland ( GBIF Secretariat, 2022).

This species is known to inhabit gardens, heathland, woodland and grassland. It has an unusual resting posture, with the front of its body elevated by its forelegs and its wings held at an angle, touching the surface it rests on. It is also identifiable by distinct physical features, such as two thin white cross-lines, dark spots in the middle of its wings, and a reddish-purple hue ( Sterling & Parsons, 2018).

The moths fly from July to August in Britain and Ireland, and are attracted to light. They mainly feed on nectar of *Calluna vulgaris*, *Tanacetum vulgare*, *Chamerion angustifolium*, *Buddleja davidii*, *Heracleum sphondylium and Jacobaea vulgaris*. The females lay their eggs in summer on the underside of leaves. The caterpillars typically feed on common agrimony ( *Agrimonia eupatoria*) and bilberries ( *Vaccinium*), as well as on various plant remains and on dry leaves of willows ( *Salix*) and oaks ( *Quercus*). This polyphagous species is not threatened in any part of its range ( Sterling & Parsons, 2018).

The genome of the Rose-flounced Tabby, *Endotricha flammealis*, was sequenced as part of the Darwin Tree of Life Project, a collaborative effort to sequence all named eukaryotic species in the Atlantic Archipelago of Britain and Ireland. A genome assembly for the Rose-flounced Tabby will give us access to valuable genomic resources that can be used to improve our understanding of lepidopteran biology, as well as provide insights into trait and function evolution in Lepidoptera superfamily ( Zhu *et al.*, 2018).

## Genome sequence report

**Figure 1.**
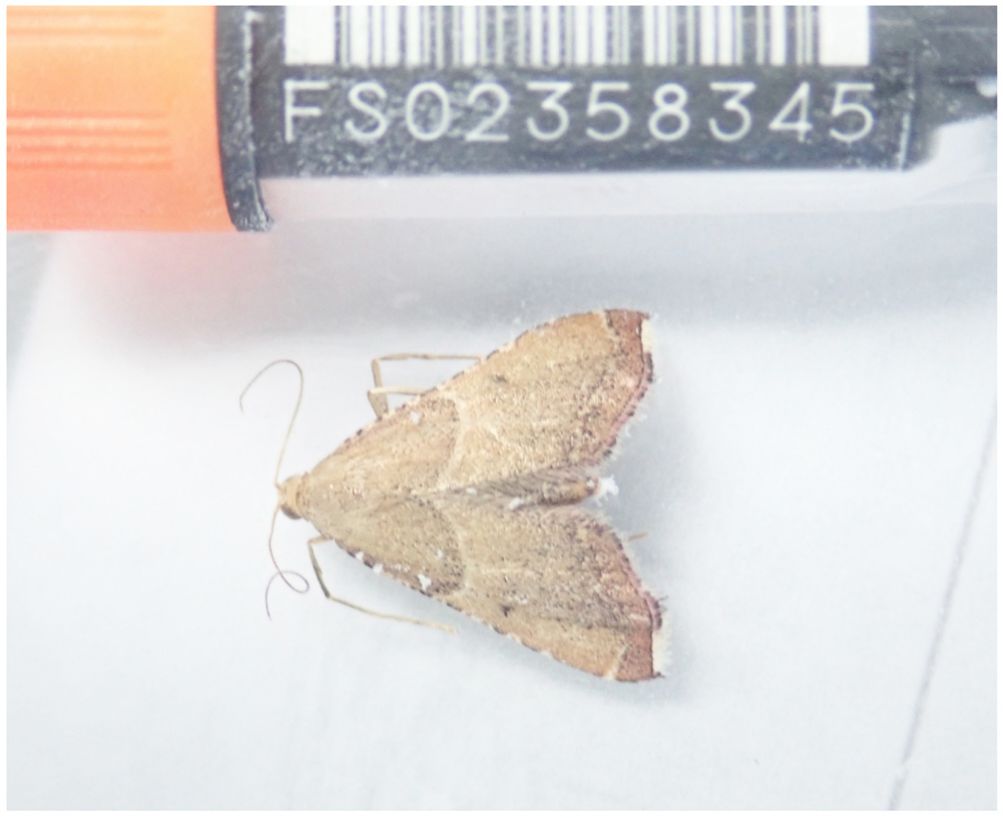
Photograph of the *Endotricha flammealis* (ilEndFlam1) specimen used for genome sequencing.

The genome was sequenced from one female *Endotricha flammealis* ( Figure 1) collected from Wytham Woods, Oxfordshire, UK (51.77, –1.33). A total of 19-fold coverage in Pacific Biosciences single-molecule HiFi long reads and 86-fold coverage in 10X Genomics read clouds were generated. Primary assembly contigs were scaffolded with chromosome conformation Hi-C data. Manual assembly curation corrected 172 missing joins or misjoins and removed 37 haplotypic duplications, reducing the assembly length by 1.71% and the scaffold number by 64.6%, and increasing the scaffold N50 by 6.33%.

**Figure 2.**
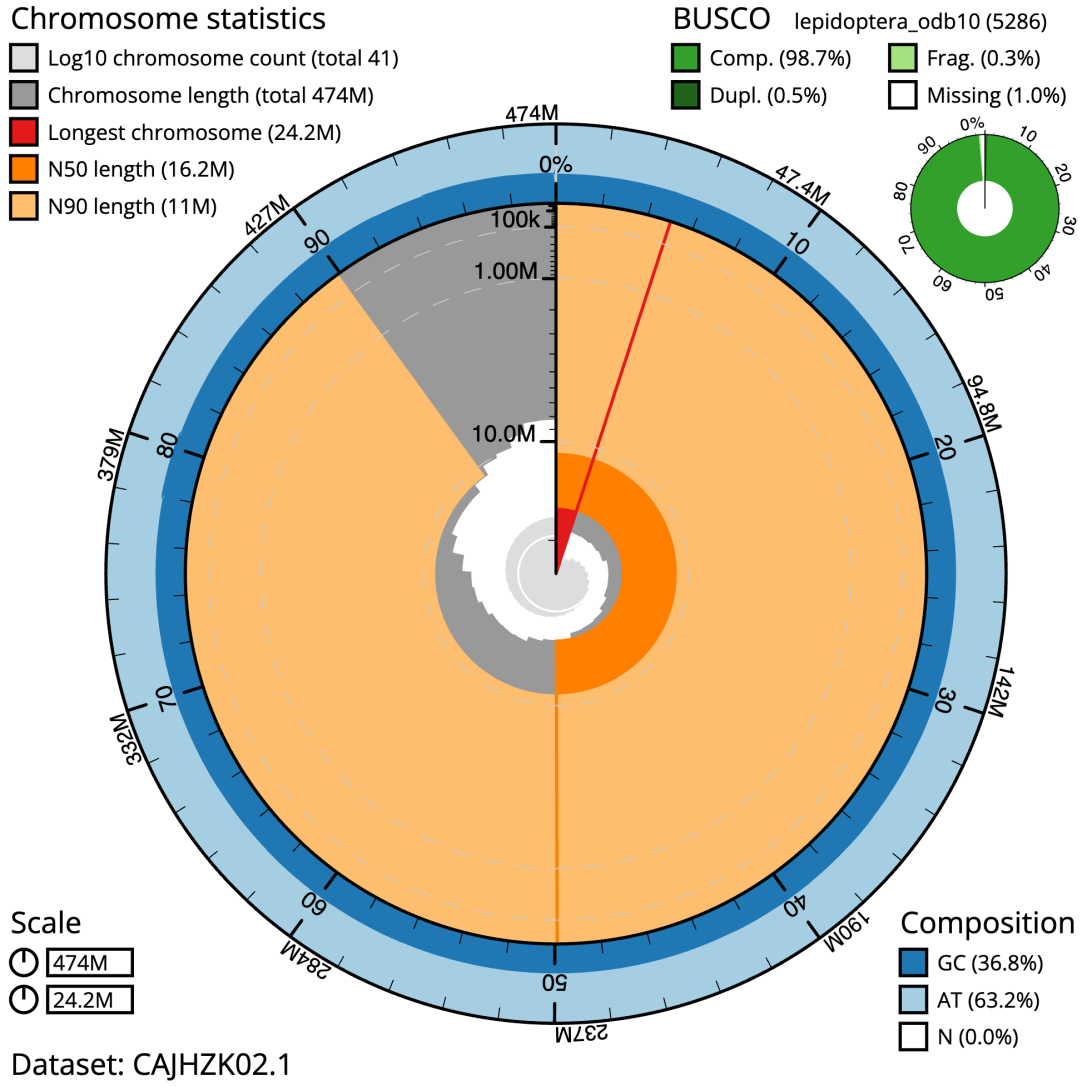
Genome assembly of *Endotricha flammealis*, ilEndFlam1.2: metrics. The BlobToolKit Snailplot shows N50 metrics and BUSCO gene completeness. The main plot is divided into 1,000 size-ordered bins around the circumference with each bin representing 0.1% of the 473,948,990 bp assembly. The distribution of scaffold lengths is shown in dark grey with the plot radius scaled to the longest scaffold present in the assembly (24,152,721 bp, shown in red). Orange and pale-orange arcs show the N50 and N90 scaffold lengths (16,153,102 and 10,965,917 bp), respectively. The pale grey spiral shows the cumulative scaffold count on a log scale with white scale lines showing successive orders of magnitude. The blue and pale-blue area around the outside of the plot shows the distribution of GC, AT and N percentages in the same bins as the inner plot. A summary of complete, fragmented, duplicated and missing BUSCO genes in the lepidoptera_odb10 set is shown in the top right. An interactive version of this figure is available at https://blobtoolkit.genomehubs.org/view/ilEndFlam1.2/dataset/CAJHZK02.1/snail.

The final assembly has a total length of 473.9 Mb in 40 sequence scaffolds with a scaffold N50 of 16.2 Mb ( Table 1). Most 99.92%) of the assembly sequence was assigned to 32 chromosomal-level scaffolds, representing 30 autosomes and the W and Z sex chromosomes. Chromosome-scale scaffolds confirmed by the Hi-C data are named in order of size ( Figure 2– Figure 5; Table 2). While not fully phased, the assembly deposited is of one haplotype. Contigs corresponding to the second haplotype have also been deposited. The mitochondrial genome was also assembled and can be found as a contig within the multifasta file of the genome submission.

**Table 1.** Genome data for *Endotricha flammealis*, ilEndFlam1.2.

Project accession data		
Assembly identifier	ilEndFlam1.2	
Species	*Endotricha flammealis*	
Specimen	ilEndFlam1	
NCBI taxonomy ID	1101095	
BioProject	PRJEB42124	
BioSample ID	SAMEA7519855	
Isolate information	ilEndFlam1, female: whole organism (DNA sequencing and Hi-C data)	
Assembly metrics *		*Benchmark*
Consensus quality (QV)	54.4	*≥ 50*
*k*-mer completeness	99.99%	*≥ 95%*
BUSCO **	C:98.7%[S:98.1%,D:0.5%], F:0.3%,M:1.0%,n:5,286	*C ≥ 95%*
Percentage of assembly mapped to chromosomes	99.92%	*≥ 95%*
Sex chromosomes	W and Z chromosomes	*localised homologous pairs*
Organelles	Mitochondrial genome assembled	*complete single alleles*
Raw data accessions		
PacificBiosciences SEQUEL II	ERR6560796	
10X Genomics Illumina	ERR6002630, ERR6002631, ERR6002633, ERR6002632	
Hi-C Illumina	ERR6002628, ERR6002629, ERR6002627	
Genome assembly		
Assembly accession	GCA_905163395.2	
*Accession of alternate haplotype*	GCA_905160925.1	
Span (Mb)	473.9	
Number of contigs	290	
Contig N50 length (Mb)	3.0	
Number of scaffolds	40	
Scaffold N50 length (Mb)	16.2	
Longest scaffold (Mb)	24.2	
Genome annotation		
Number of protein-coding genes	17,578	
Number of gene transcripts	17,578	

* Assembly metric benchmarks are adapted from column VGP-2020 of “Table 1: Proposed standards and metrics for defining genome assembly quality” from ( Rhie *et al.*, 2021). ** BUSCO scores based on the lepidoptera_odb10 BUSCO set using v5.3.2. C = complete [S = single copy, D = duplicated], F = fragmented, M = missing, n = number of orthologues in comparison. A full set of BUSCO scores is available at https://blobtoolkit.genomehubs.org/view/ilEndFlam1.2/dataset/CAJHZK02.1/busco.

**Figure 3.**
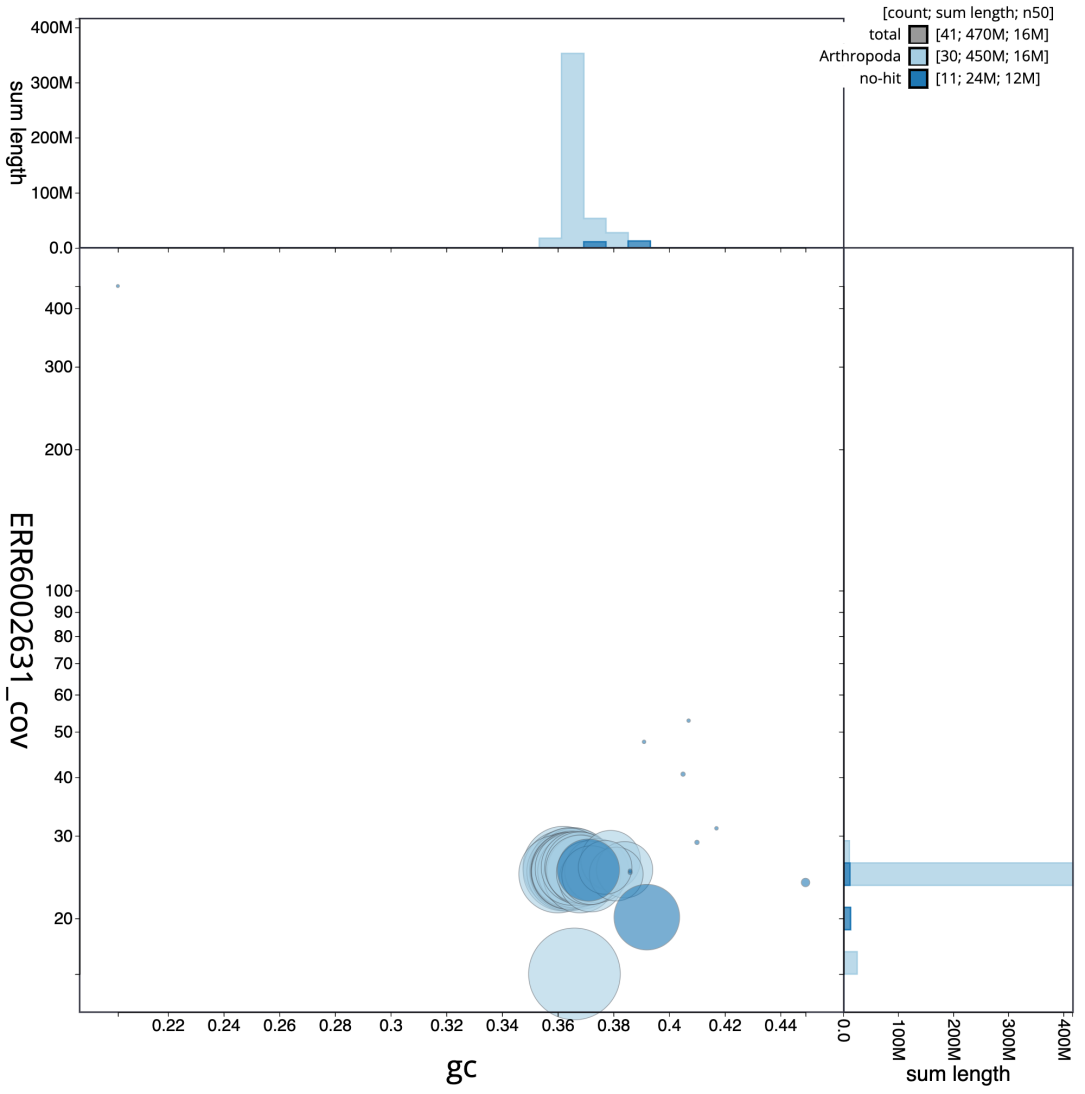
Genome assembly of *Endotricha flammealis*, ilEndFlam1.2: BlobToolKit GC-coverage plot. Scaffolds are coloured by phylum. Circles are sized in proportion to scaffold length. Histograms show the distribution of scaffold length sum along each axis. An interactive version of this figure is available at https://blobtoolkit.genomehubs.org/view/ilEndFlam1.2/dataset/CAJHZK02.1/blob.

**Figure 4.**
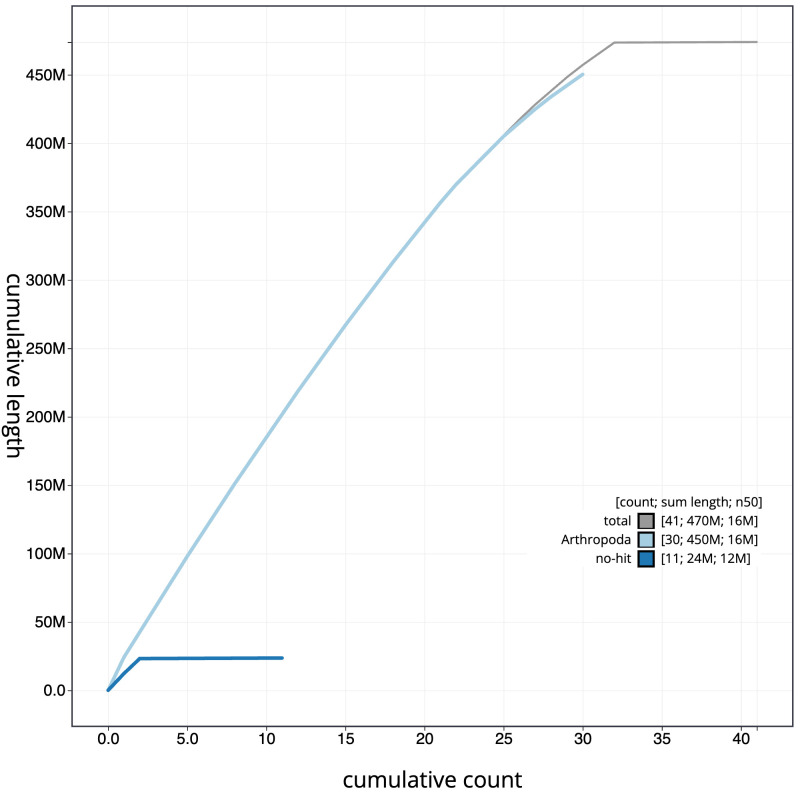
Genome assembly of *Endotricha flammealis*, ilEndFlam1.2: BlobToolKit cumulative sequence plot. The grey line shows cumulative length for all scaffolds. Coloured lines show cumulative lengths of scaffolds assigned to each phylum using the buscogenes taxrule. An interactive version of this figure is available at https://blobtoolkit.genomehubs.org/view/ilEndFlam1.2/dataset/CAJHZK02.1/cumulative.

**Figure 5.**
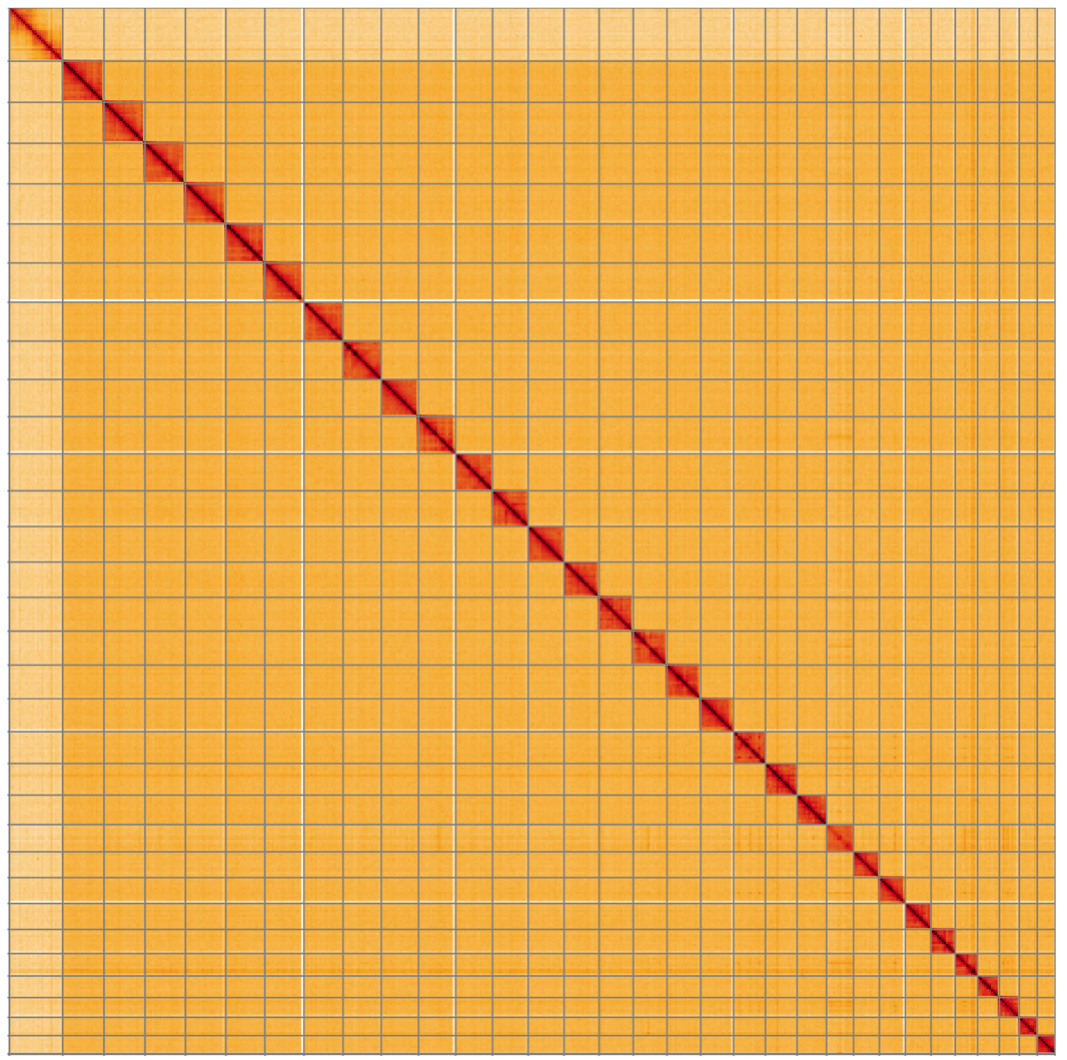
Genome assembly of *Endotricha flammealis*, ilEndFlam1.2: Hi-C contact map of the ilEndFlam1.2 assembly, visualised using HiGlass. Chromosomes are shown in order of size from left to right and top to bottom. An interactive version of this figure may be viewed at https://genome-note-higlass.tol.sanger.ac.uk/l/?d=JxsPDaCSQIGNGfqEzxAHdQ.

**Table 2.** Chromosomal pseudomolecules in the genome assembly of *Endotricha flammealis*, ilEndFlam1.

INSDC accession	Chromosome	Length (Mb)	GC%
LR990853.1	1	18.65	36.0
LR990854.1	2	18.56	36.0
LR990855.1	3	18.37	36.5
LR990856.1	4	18.19	36.5
LR990857.1	5	17.71	36.5
LR990858.1	6	17.69	36.5
LR990859.1	7	17.65	36.5
LR990860.1	8	17.31	36.0
LR990861.1	9	16.91	36.5
LR990862.1	10	16.8	37.0
LR990863.1	11	16.73	36.5
LR990864.1	12	16.16	36.5
LR990865.1	13	16.15	36.5
LR990866.1	14	15.94	36.5
LR990867.1	15	15.39	36.5
LR990868.1	16	15.32	37.0
LR990869.1	17	15.25	36.5
LR990870.1	18	14.86	36.5
LR990871.1	19	14.46	37.0
LR990872.1	20	14.27	37.0
LR990873.1	21	13.38	37.0
LR990874.1	W	12.27	39.0
LR990875.1	22	11.72	37.0
LR990876.1	23	11.72	37.5
LR990877.1	24	11.7	37.0
LR990878.1	25	10.97	37.0
LR990879.1	26	10.11	38.0
LR990880.1	27	9.81	37.0
LR990881.1	28	9.01	38.5
LR990882.1	29	8.19	38.0
LR990883.1	30	8.17	37.5
LR990852.1	Z	24.15	36.5
LR990884.2	MT	0.02	20.5

The estimated Quality Value (QV) of the final assembly is 54.4 with *k*-mer completeness of 99.99%, and the assembly has a BUSCO v5.3.2 completeness of 98.7% (single = 98.1%, duplicated = 0.5%), using the lepidoptera_odb10 reference set ( *n* = 5,286).

Metadata for specimens, spectral estimates, sequencing runs, contaminants and pre-curation assembly statistics can be found at https://links.tol.sanger.ac.uk/species/1101095.

## Genome annotation report

The *Endotricha flammealis* genome assembly (GCA_905163395.2) was annotated using the Ensembl rapid annotation pipeline ( Table 1; https://rapid.ensembl.org/Endotricha_flammealis_GCA_905163395.2/Info/Index). The resulting annotation includes 17,781 transcribed mRNAs from 17,578 protein-coding genes.

## Methods

### Sample acquisition and nucleic acid extraction

A female *Endotricha flammealis* (specimen ID Ox000062, individual ilEndFlam1) was collected from Wytham Woods, Oxfordshire (biological vice-country Berkshire), UK (latitude 51.77, longitude –1.33) on 2019-07-17 using a light trap. The specimen was collected and identified by Douglas Boyes (University of Oxford) and was snap-frozen on dry ice.

DNA was extracted at the Tree of Life laboratory, Wellcome Sanger Institute (WSI). The ilEndFlam1 sample was weighed and dissected on dry ice with tissue set aside for Hi-C sequencing. Tissue from the whole organism was disrupted using a Nippi Powermasher fitted with a BioMasher pestle. High molecular weight (HMW) DNA was extracted using the Qiagen MagAttract HMW DNA extraction kit. Low molecular weight DNA was removed from a 20 ng aliquot of extracted DNA using the 0.8X AMpure XP purification kit prior to 10X Chromium sequencing; a minimum of 50 ng DNA was submitted for 10X sequencing. HMW DNA was sheared into an average fragment size of 12–20 kb in a Megaruptor 3 system with speed setting 30. Sheared DNA was purified by solid-phase reversible immobilisation using AMPure PB beads with a 1.8X ratio of beads to sample to remove the shorter fragments and concentrate the DNA sample. The concentration of the sheared and purified DNA was assessed using a Nanodrop spectrophotometer and Qubit Fluorometer and Qubit dsDNA High Sensitivity Assay kit. Fragment size distribution was evaluated by running the sample on the FemtoPulse system.

### Sequencing

Pacific Biosciences HiFi circular consensus and 10X Genomics read cloud DNA sequencing libraries were constructed according to the manufacturers’ instructions. DNA sequencing was performed by the Scientific Operations core at the WSI on Pacific Biosciences SEQUEL II (HiFi) and HiSeq X Ten (10X) instruments. Hi-C data were also generated from tissue of ilEndFlam1 using the Arima2 kit and sequenced on the HiSeq X Ten instrument.

### Genome assembly, curation and evaluation

Assembly was carried out with Hifiasm ( Cheng *et al.*, 2021) and haplotypic duplication was identified and removed with purge_dups ( Guan *et al.*, 2020). One round of polishing was performed by aligning 10X Genomics read data to the assembly with Long Ranger ALIGN, calling variants with FreeBayes ( Garrison & Marth, 2012). The assembly was then scaffolded with Hi-C data ( Rao *et al.*, 2014) using SALSA2 ( Ghurye *et al.*, 2019). The assembly was checked for contamination and corrected using the gEVAL system ( Chow *et al.*, 2016) as described previously ( Howe *et al.*, 2021). Manual curation was performed using gEVAL, HiGlass ( Kerpedjiev *et al.*, 2018) and Pretext ( Harry, 2022). The mitochondrial genome was assembled using MitoHiFi ( Uliano-Silva *et al.*, 2023), which runs MitoFinder ( Allio *et al.*, 2020) or MITOS ( Bernt *et al.*, 2013) and uses these annotations to select the final mitochondrial contig and to ensure the general quality of the sequence.

A Hi-C map for the final assembly was produced using bwa-mem2 ( Vasimuddin *et al.*, 2019) in the Cooler file format ( Abdennur & Mirny, 2020). To assess the assembly metrics, the *k*-mer completeness and QV consensus quality values were calculated in Merqury ( Rhie *et al.*, 2020). This work was done using Nextflow ( Di Tommaso *et al.*, 2017) DSL2 pipelines “sanger-tol/readmapping” ( Surana *et al.*, 2023a) and “sanger-tol/genomenote” ( Surana *et al.*, 2023b). The genome was analysed within the BlobToolKit environment ( Challis *et al.*, 2020) and BUSCO scores ( Manni *et al.*, 2021; Simão *et al.*, 2015) were calculated.

Table 3 contains a list of relevant software tool versions and sources.

**Table 3.** Software tools: versions and sources.

Software tool	Version	Source
BlobToolKit	4.1.7	https://github.com/blobtoolkit/blobtoolkit
BUSCO	5.3.2	https://gitlab.com/ezlab/busco
FreeBayes	1.3.1-17-gaa2ace8	https://github.com/freebayes/freebayes
gEVAL	N/A	https://geval.org.uk/
Hifiasm	0.7	https://github.com/chhylp123/hifiasm
HiGlass	1.11.6	https://github.com/higlass/higlass
Long Ranger ALIGN	2.2.2	https://support.10xgenomics.com/genome-exome/software/pipelines/latest/advanced/other-pipelines
Merqury	MerquryFK	https://github.com/thegenemyers/MERQURY.FK
MitoHiFi	2	https://github.com/marcelauliano/MitoHiFi
PretextView	0.2	https://github.com/wtsi-hpag/PretextView
purge_dups	1.2.3	https://github.com/dfguan/purge_dups
SALSA	2.2	https://github.com/salsa-rs/salsa
sanger-tol/genomenote	v1.0	https://github.com/sanger-tol/genomenote
sanger-tol/readmapping	1.1.0	https://github.com/sanger-tol/readmapping/tree/1.1.0

### Genome annotation

The BRAKER2 pipeline ( Brůna *et al.*, 2021) was used in the default protein mode to generate annotation for the *Endotricha flammealis* assembly (GCA_905163395.2) in Ensembl Rapid Release.

### Wellcome Sanger Institute – Legal and Governance

The materials that have contributed to this genome note have been supplied by a Darwin Tree of Life Partner. The submission of materials by a Darwin Tree of Life Partner is subject to the **‘Darwin Tree of Life Project Sampling Code of Practice’**, which can be found in full on the Darwin Tree of Life website here. By agreeing with and signing up to the Sampling Code of Practice, the Darwin Tree of Life Partner agrees they will meet the legal and ethical requirements and standards set out within this document in respect of all samples acquired for, and supplied to, the Darwin Tree of Life Project.

Further, the Wellcome Sanger Institute employs a process whereby due diligence is carried out proportionate to the nature of the materials themselves, and the circumstances under which they have been/are to be collected and provided for use. The purpose of this is to address and mitigate any potential legal and/or ethical implications of receipt and use of the materials as part of the research project, and to ensure that in doing so we align with best practice wherever possible. The overarching areas of consideration are:

•   Ethical review of provenance and sourcing of the material

•   Legality of collection, transfer and use (national and international) 

Each transfer of samples is further undertaken according to a Research Collaboration Agreement or Material Transfer Agreement entered into by the Darwin Tree of Life Partner, Genome Research Limited (operating as the Wellcome Sanger Institute), and in some circumstances other Darwin Tree of Life collaborators.

## Data Availability

European Nucleotide Archive: *Endotricha flammealis* (rose-flounced tabby). Accession number PRJEB42124; https://identifiers.org/ena.embl/PRJEB42124. ( Wellcome Sanger Institute, 2022) The genome sequence is released openly for reuse. The *Endotricha flammealis* genome sequencing initiative is part of the Darwin Tree of Life (DToL) project. All raw sequence data and the assembly have been deposited in INSDC databases. Raw data and assembly accession identifiers are reported in Table 1.

## Author information

Members of the University of Oxford and Wytham Woods Genome Acquisition Lab are listed here: https://doi.org/10.5281/zenodo.4789928.

Members of the Darwin Tree of Life Barcoding collective are listed here: https://doi.org/10.5281/zenodo.4893703.

Members of the Wellcome Sanger Institute Tree of Life programme are listed here: https://doi.org/10.5281/zenodo.4783585.

Members of Wellcome Sanger Institute Scientific Operations: DNA Pipelines collective are listed here: https://doi.org/10.5281/zenodo.4790455.

Members of the Tree of Life Core Informatics collective are listed here: https://doi.org/10.5281/zenodo.5013541.

Members of the Darwin Tree of Life Consortium are listed here: https://doi.org/10.5281/zenodo.4783558.
