# Supplementary material for: The genome sequence of the Rose-flounced Tabby, Endotricha flammealis (Denis & Schiffermüller, 1775)
Source: Wellcome Open Res. 2023 Nov 13;8:516. [Version 1] doi: 10.12688/wellcomeopenres.19923.1 (PMC11289590; doi:10.12688/wellcomeopenres.19923.1)
Supplement: Peer review history

# Reviewer report: Reviewer response for version 1

**DOI:** 10.21956/wellcomeopenres.22062.r85822

**Published:** 2024-07-30

**Authors:** Marylin Miga

**Affiliations:** 1 Department of Biosciences, Faculty of Science, Universiti Teknologi Malaysia, Johor Bahru, Johor, 81310, Malaysia

The paper discusses the genome sequencing and assembly of the Rose-flounced Tabby moth, *Endotricha flammealis*. The genome was sequenced using high molecular weight DNA extracted from the whole organism which resulted in a 473.9 megabases in length. The assembly, curated and evaluated using various tools, resulted in 32 chromosomal pseudomolecules and the identification of 17,578 protein-coding genes. The genome sequence is openly available for further research and provides valuable insights into lepidopteran biology. The report is quite straightforward to understand and provides valuable data to further understand lepidopteran biology.

 However, for Figure 1, I recommend the authors put a scale to indicate the size/length of the species.

Are sufficient details of methods and materials provided to allow replication by others?

Yes

Is the rationale for creating the dataset(s) clearly described?

Yes

Are the datasets clearly presented in a useable and accessible format?

Yes

Are the protocols appropriate and is the work technically sound?

Yes

Reviewer Expertise:

entomology, biodiversity, bioinformatics

I confirm that I have read this submission and believe that I have an appropriate level of expertise to confirm that it is of an acceptable scientific standard.

# Reviewer report: Reviewer response for version 1

**DOI:** 10.21956/wellcomeopenres.22062.r88713

**Published:** 2024-07-15

**Authors:** Kay Lucek

**Affiliations:** 1 University of Basel, Basel, Switzerland

The authors present the chromosome level genome assembly of a male specimen of the Rose-flounced Tabby moth *Endotricha flammealis*. The assembly consists of 32 chromosomes including both sex chromosomes. The assembly is highly complete as revealed by the high BUSCO score but not fully phased. Sequencing and genome assembly follow the current state of the art and use established methods.

 Overall, the presented assembly will be of great value to study genome architecture as well as genome size evolution in Lepidoptera. It is very interesting to see that a 19X long read coverage is enough to resolve a chromosome level assembly in this holocentric species group. Although I was wondering if this could explain the additional larger scaffolds that were not part of the chromosome assembly.

Are sufficient details of methods and materials provided to allow replication by others?

Yes

Is the rationale for creating the dataset(s) clearly described?

Yes

Are the datasets clearly presented in a useable and accessible format?

Yes

Are the protocols appropriate and is the work technically sound?

Yes

Reviewer Expertise:

Speciation genomics

I confirm that I have read this submission and believe that I have an appropriate level of expertise to confirm that it is of an acceptable scientific standard.
